# Identification of Gastritis Subtypes by Convolutional Neuronal Networks on Histological Images of Antrum and Corpus Biopsies

**DOI:** 10.3390/ijms21186652

**Published:** 2020-09-11

**Authors:** Georg Steinbuss, Katharina Kriegsmann, Mark Kriegsmann

**Affiliations:** 1 Department of Hematology, Oncology and Rheumatology, University Hospital Heidelberg, 69120 Heidelberg, Germany; georg.steinbuss@med.uni-heidelberg.de (G.S.); katharina.kriegsmann@med.uni-heidelberg.de (K.K.); 2 Institute of Pathology, University Hospital Heidelberg, 69120 Heidelberg, Germany

**Keywords:** deep learning, digital image analysis, convolutional neural networks, artificial intelligence

## Abstract

Background: Gastritis is a prevalent disease and commonly classified into autoimmune (A), bacterial (B), and chemical (C) type gastritis. While the former two subtypes are associated with an increased risk of developing gastric intestinal adenocarcinoma, the latter subtype is not. In this study, we evaluated the capability to classify common gastritis subtypes using convolutional neuronal networks on a small dataset of antrum and corpus biopsies. Methods: 1230 representative 500 × 500 µm images of 135 patients with type A, type B, and type C gastritis were extracted from scanned histological slides. Patients were allocated randomly into a training set (60%), a validation set (20%), and a test set (20%). One classifier for antrum and one classifier for corpus were trained and optimized. After optimization, the test set was analyzed using a joint result from both classifiers. Results: Overall accuracy in the test set was 84% and was particularly high for type B gastritis with a sensitivity of 100% and a specificity of 93%. Conclusions: Classification of gastritis subtypes is possible using convolutional neural networks on a small dataset of histopathological images of antrum and corpus biopsies. Deep learning strategies to support routine diagnostic pathology merit further evaluation.

## 1. Introduction

Chronic gastritis is a very prevalent disease and has an estimated prevalence of more than half of the world’s population [1]. The most commonly used histological classification system for gastritis is the Sydney classification introduced in 1990 [2], updated in 1994, and modified in 2005 [3]. It provides a consensus terminology and evaluates the degree of active and chronic inflammation as well as the presence of *Helicobacter pylori*, atrophy, and intestinal metaplasia in four increments: normal, mild, moderate, and severe. Other systems such as the Operative Link on Gastritis Assessment (OLGA) and the Operative Link on Gastritis Assessment based on Intestinal Metaplasia (OLGIM) systems were proposed for staging of atrophy and intestinal metaplasia and were recently validated to reliably predict gastric cancer risk [4,5].

Besides these common classification systems, a more simplistic and older classification considering only the most common etiologies into autoimmune (A), bacterial (B), and chemical (C) gastritis is still in use. These classification systems aim to inform the clinician not only about the underlying etiology but also about the associated risk for the development of gastric cancer [5].

Type A gastritis is the least common subtype with a reported prevalence rate of about 1–2% and is characterized by an autoimmune T-cell mediated destruction of oxyntic glands with progressive atrophy and intestinal or pancreatic acinar metaplasia of the gastric corpus mucosa [6,7]. Specifically, in the earlier course of the disease, a patchy full thickness and bottom predominant lymphoplasmacytic infiltrate is observed in the lamina propria. Often in the later course of the disease, a linear or nodular enterochromaffin-like cell hyperplasia is evident. The risk for carcinoid tumors and intestinal type adenocarcinoma is increased [8,9].

Type B gastritis is the most common subtype; it affects about two thirds of the world’s population and is one of the most common inflammatory diseases in humans. Its prevalence is highly variable with lower rates in industrialized countries and children. It is caused mostly (over 99%) by the bacterium *Helicobacter pylori* [10,11]. Histologically, non-atrophic and atrophic patterns may be observed [12]. Common histological features are neutrophilic granulocytes in the lamina propria and the epithelium, a lymphoplasmacellular infiltrate, which is often superficial in the corpus, as well as lymphoid follicles with germinal centers. Intestinal metaplasia of antral mucosa and atrophy of corpus mucosa are frequently observed. Pangastritis and corpus-predominant gastritis are common patterns in type B gastritis and are believed to be predisposing conditions to atrophy [13]. The identification of atrophy is particularly important, as a large body of evidence supports that atrophy is the single most important risk factor for intestinal-type gastric cancer [13,14]. Therefore, pangastritis and corpus-predominant B gastritis have also been referred as high-risk gastritis for the development of intestinal-type gastric cancer [15].

Type C gastritis is the second most common subtype of gastritis and can be caused by various agents, among which alcohol and non-steroidal anti-inflammatory drugs are the most common. Histological changes can vary and include edema, foveolar hyperplasia of antral mucosa, a mild chronic inflammation, vascular congestion, reactive epithelial changes, and smooth muscle hyperplasia in the lamina propria. This type of gastritis is not generally associated with an increased risk of gastric cancer.

In the past decade, significant advances have been made in applying convolutional neural networks (CNN) to histological scans, particularly to classify neoplastic diseases [16]. Non-neoplastic diseases were rarely analyzed by machine learning and for the classification of gastritis only one study has been conducted to the best of our knowledge [17,18]. Classification of gastritis presents a particular challenge, as images from both antrum and corpus have to be taken into account.

In this study, we applied CNNs to a small dataset and evaluated their capability to classify the most common gastritis subtypes A, B, and C.

## 2. Results

### 2.1. Patient Cohort, Annotation, Image Patches Extraction and Subset Analysis

**Figure 1:**
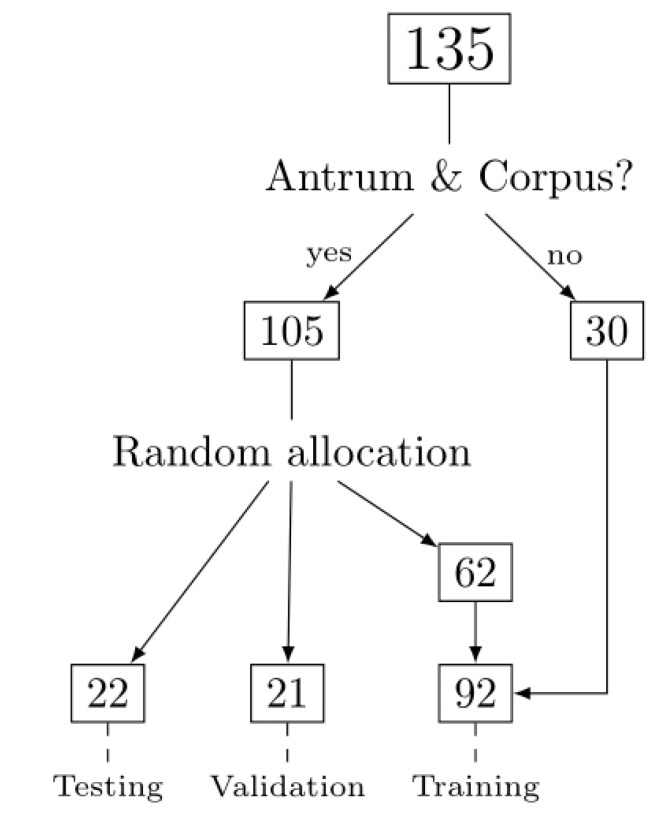
Flowchart of allocation of cases. All 135 cases were screened for the presents of antrum and corpus mucosa. Cases were randomly assigned into a training set, a validation set, and a test set. Cases with tissue samples from antrum or corpus mucosa only, were always added to the training set and not randomly assigned.

Cases from A (*n* = 49), B (*n* = 39), and C (*n* = 47) gastritis were successfully identified, retrieved, stained, and scanned. The regions of interest were annotated and divided into image patches which were labeled according to the diagnosis as low inflammation (LI) and severe inflammation (SI) in the antrum and as SI, LI, and A gastritis in the corpus. Identification of the respective gastritis related regions resulted in a total of 1230 extracted 500 × 500 µm (1973 × 1973 px) image patches. With 124 cases, tissue from the antrum was provided and with 116 from the corpus. Therefore, a complete antrum/corpus set was not available with every case. The workflow and allocation of cases among the different sets is displayed in Figure 1.

**Figure 2:**
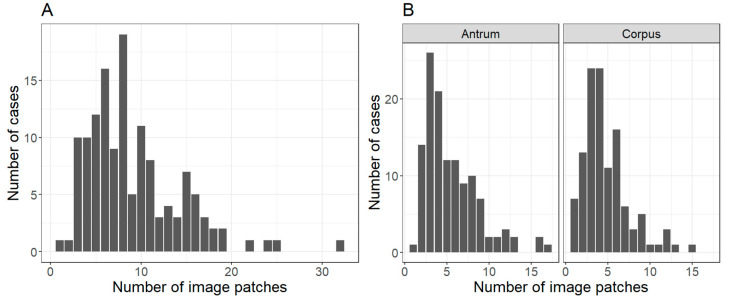
Number of extracted image patches. The figure shows the number of extracted image patches (**A**) per case overall and (**B**) per case and tissue type.

The number of image patches extracted per case and tissue type is displayed in Figure 2.

From the total number of 135 cases, in 105 cases, tissue from the antrum and corpus was available, while in 30 patients, only one tissue type was available. The 105 patients with antrum and corpus were randomly allocated in a training set, a validation set, and test set (60%, 20%, and 20% of patients, respectively). Following this allocation, cases with either only antrum or corpus were assigned (i.e., incomplete antrum/corpus set) to the training data (Table 1).

### 2.2. CNN Training, Validation, and Model Selection

Different models were trained for antrum and corpus, respectively. In both cases, the Xception CNN architecture was used and optimized according to a set of different hyperparameters: dropout rate before the last logistic layer (0.0, 0.2, and 0.5), L2 regularization (0.0, 2 × 10^−5^), and learning rate (1 × 10^−4^, 1 × 10^−5^, 1 × 10^−6^, 1 × 10^−7^, 1 × 10^−8^). Each model was trained for 100 epochs with a batch size of eight. For the input of the CNN, the image patches were compressed to 299 × 299 px. A summary of the validation Area Under the receiver operator characteristics Curve (AUC) along with the degree of overfitting for each trained model is shown in Supplementary Figure S1. The final models for antrum and corpus are those models (among the differently parametrized models) that yield the highest validation AUC.

#### 2.2.1. Antrum Classification Model

**Figure 3:**
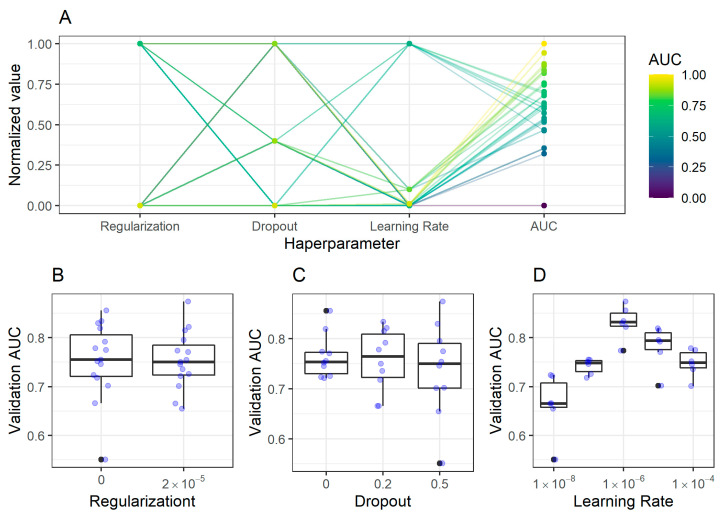
Impact of hyperparameters on the validation Area Under the receiver operator characteristics Curve (AUC) in classification of antrum image patches. (**A**) The coordinates plot shows the dependency of hyperparameters and resulting AUC for different trained models shown as dots. The plot shows the normalized values of each parameter, e.g., while the actual values for dropout were 0 and 0.5, these values are shown in the plot as 0 and 1 respectively. The same holds for the AUC value. This normalization improves visualization in the coordinates plot. While (**B**) regularization and (**C**) dropout had almost no consistent impact on the validation AUC of antrum classification models, the (**D**) learning rate had an impact, displaying the highest AUC at 1 × 10^−6^.

As shown in Figure 3, only the learning rate had a significant impact on the AUC of the antrum classifier. The final model had the following configuration: a dropout rate of 0.5, regularization of 2 × 10^−5^, and learning rate of 1 × 10^−6^.

#### 2.2.2. Corpus Classification Model

**Figure 4:**
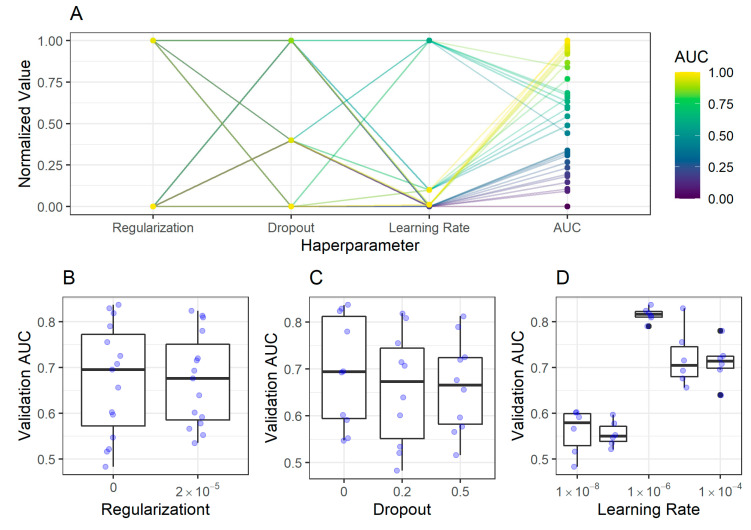
Impact of hyperparameters on the validation AUC in classification of corpus image patches. (**A**) The coordinates plot shows the dependency of hyperparameters and resulting AUC for different trained models shown as dots. While the (**B**) regularization and (**C**) dropout had almost no consistent impact on the validation AUC of corpus image patches, the (**D**) learning rate had an impact, displaying the highest AUC at 1 × 10−6.

Similarly to antrum classification models, only the modification of the learning rate had a significant impact on the validation AUC for the corpus classification models. The final corpus classifier model had the following configuration: dropout rate 0, regularization 0, and learning rate 1 × 10^−6^ (Figure 4).

### 2.3. Image Patch Prediction Results for the Validation and Test Set

For each image patch, the predicted class (LI, SI, or A gastritis with patches from the corpus) was the class with the highest prediction probability of the respective classifier. Applying this to the implemented antrum classifier in the validation set, an overall accuracy of 0.77 (95% confidence interval [CI] 0.68–0.84) was achieved. For the corpus classifier, an overall accuracy of 0.67 (95% CI 0.55–0.77) was reached in the validation set. A detailed confusion matrix and statistics for the validation set are given in Table 2.

An independent test set is of outstanding importance for the evaluation of the established classification models since the best models were chosen based on their AUC with the validation data. Applying the implemented antrum classifier on the test set an overall accuracy of 0.85 (95% CI 0.77–0.91) was reached. The corpus classifier achieved an overall accuracy of 0.56 (95% CI 0.46–0.66). A detailed confusion matrix and statistics for the test set are given in Table 3. Examples of misclassified image patches are given in Supplementary Figure S2.

**Figure 5:**
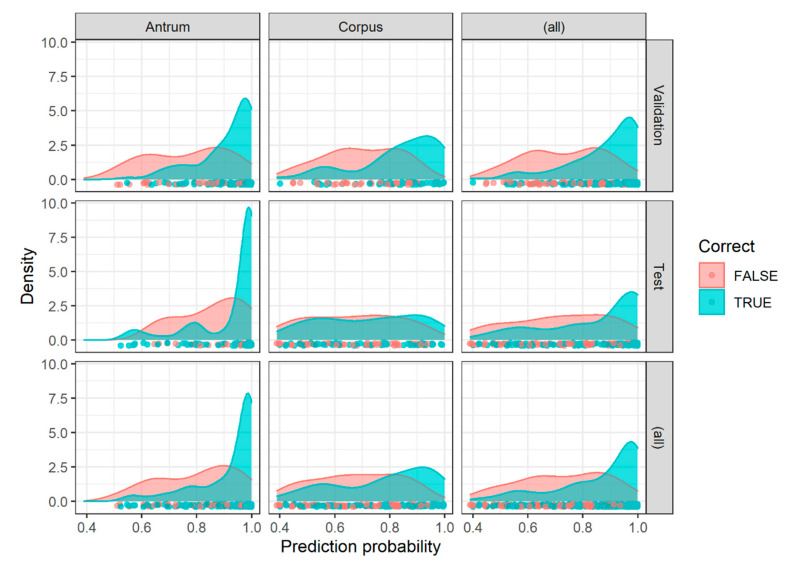
Classification result according to the prediction probability. The classification results (correct yes versus not) of image patches from the antrum validation set, corpus validation set, antrum test set, and corpus test set are shown according to their prediction probability along with marginal summaries (all).

Generally, as shown in Figure 5, image patches showing a higher prediction probability for a certain class were more likely to be classified correctly. This was particularly true for the antrum classifier.

To establish a diagnosis, a joint evaluation of the antrum and corpus image patch classification results on a patient level is necessary. As a first step, a majority vote on the antrum and classification level was established, i.e., only the predicted classes with the most predictions were kept. For example, if for a patient X, the corpus classifier predicted one corpus image patch with LI and three image patches with SI, only the SI prediction was kept for this patient and the respective corpus. Applying this procedure, 5 of 22 patients in the test set showed ties, i.e., equal number of images patches predicted for two classes, and therefore no possible majority vote. The overall diagnosis was based on the overall antrum and corpus prediction as described in the methods section. For the 17 patients in the test set without ties, an overall classification accuracy for the three gastritis subtypes of 0.88 (95% CI 0.64–0.96) was reached (Table 4).

The five remaining patients with ties on the image patch level were evaluated separately. As the majority vote was not possible in this situation, two possible classification results were accepted on the antrum or corpus level, respectively, and the overall diagnosis of the gastritis type was established twice. Interestingly, in two of the five patients, the tie had no influence on the final gastritis diagnosis, as the second evaluation site (corpus or antrum, respectively) already established the diagnosis. Exemplarily, SI was predicted in the antrum biopsy, while in the corpus, 50% of image patches voted for SI and 50% for LI. Therefore, the final diagnosis (B gastritis) would result from the antrum classifier (voted for SI) irrespective of the corpus classifier.

## 3. Discussion

Gastritis is one of the most common inflammatory disorders. Specific subtypes of gastritis have been defined that are associated with a different risk of gastric cancer [19]. In the present study, we have analyzed the possibility to use CNNs for the classification of gastritis and the identification of high-risk gastritis subtypes on a small dataset.

Small datasets pose a particular challenge, as there is a significant risk of overfitting and insufficient generalization capability: with a rather low variation of examples in the training data, the CNN is likely to focus on features of these examples that do not generalize to external examples [20]. In the following section, we discuss measures that have been taken to face this problem, including the proportions for the sets, choice of the CNN architecture, the use of image augmentation, and the optimization strategy.

The creation of image patches from a scanned histological slide is necessary, as CNN can process only limited image sizes [21]. The separation of 60%, 20%, and 20% for the training, validation, and test sets was arbitrary, and there is currently no established gold standard [21,22,23,24,25]. A higher proportion of cases in the training set is expected to result in a more robust model, but the data in the validation and test cohort would possibly not be representative. Nonetheless, separation into the three sets is mandatory, as during hyperparameter tuning (specifically the selection of optimal models), information from the training set migrates into the validation set. Thus, the capacity of the model must be tested on a separate test set.

Various CNNs are available for the classification of images. Commonly used CNNs for histological and cytological images are VGG16 [16,26,27], InceptionV3 [28,29], and InceptionResNetV2 [30]. Some of these CNNs are rather large (VGG16, InceptionResNetV2) and achieve high accuracies with large training datasets. In our case, the use of such large networks was not appropriate as our dataset is very small and the risk of overfitting would have been high [31]. A possible solution is to use a rather lightweight CNN such as Xception, which is an adaptation from the Inception architecture, where the Inception modules have been replaced with depthwise separable convolutions [32,33]. The Xception architecture outperformed the InceptionV3 network on the ImageNet dataset and was previously used to successfully classify clinical images of skin pathologies and computed tomography images [34,35]. Therefore, this network architecture seemed most appropriate for our purpose.

Image augmentation is a common strategy when working with limited training data and aims to increase the data size in the training set without acquiring new images [36]. During the process, the images are duplicated and shown again to the network with some kind of variation. For example, an image is turned by 90 or 180 degrees. As we found a moderate overfitting during training, we also tested image augmentation. However, the effect was only minimal, which is probably due to the fact that the differences inherent to the images are rather large, and the effect of showing the same image only slightly altered did not force the CNNs to learn generalizable features of the different classes. We are almost certain that image augmentation in combination with a larger dataset would be a good strategy to also tackle overfitting in images of gastritis.

Optimization of a CNN during the training process is necessary to achieve high classification accuracies [37,38]. Hyperparameters may be optimized which often includes testing various hyperparameter combinations, a process that may be limited by the computational power available [39,40]. With respect to the available computational power, we decided to optimize regularization, dropout, and learning rate. Currently, there is no established standard for the optimization process of a CNN model [41,42,43,44].

Moreover, it was clear that *Helicobacter pylori* itself cannot be identified by the deep learning algorithm, as the organism is too small to be reliably identified on extracted image patches by the human eye. Therefore, the inflammatory pattern had to be considered as a surrogate for type A and type B gastritis. Type B gastritis exhibits a characteristic inflammatory pattern which is commonly superficial and band-like in antrum and corpus, different from type A gastritis, where the inflammation is commonly pronounced in corpus mucosa and located more basally, and type C gastritis, where inflammation is often much less prominent. The approach to take the inflammatory pattern as a surrogate was also chosen in a previous study using deep learning for the classification of gastritis [18]. The difference in our study is that in the respective study, a larger dataset was available and normal (non-inflamed) gastric tissue, type B, and type C gastritis were analyzed.

We anticipated the problem that the localization of the inflammatory infiltrate is important for the classification by taking rather large images of 500 × 500 µm. Taking smaller images (e.g., 100 × 100 µm corresponding to 395 × 395 px), would have resulted in a very small field of view and subsequently to a high change of missing the specific features of the respective gastritis subtype at our scanning magnification (400×). The downside of this approach was the relatively low number of images obtained per patient. As our dataset was rather small, we could not apply quality measurements as previously proposed by our group [16].

Another important aspect in our study was that both images from antrum and corpus had to be considered to achieve a reliable result. In this regard, it is important to note that type A gastritis can only be diagnosed on corpus biopsies and type C gastritis cannot be diagnosed on corpus biopsies. Therefore, we trained two classifiers: one corpus classifier considering type A gastritis, low and severe inflammation, as well as an antrum classifier considering low and severe inflammation. A combination of the classification output was considered in the final diagnostic result. While studies combining multiple CNN architectures exist, we are not aware of a study using this innovative approach to combine the classification results from different anatomic regions to achieve a final classification result on histopathological images [45]. In principle, this approach allows the identification of severe pangastritis and corpus-predominant type B gastritis, which are believed to be associated with a high risk for the development of intestinal type adenocarcinoma. However, the number of cases in our study was too limited to draw any final conclusions, whether our approach can correctly classify high-risk gastritis patterns.

Our study has several limitations including mainly the sample size and the number of included subtypes of gastritis. We examined a total of 135 cases per gastritis subtype. Based on the random separation into training, validation, and test sets, only a few more than 70 to 80 patients (different case numbers for antrum and corpus) were included in the training set. Based on the limited sample size, it is remarkable that our classifier was able to achieve a classification accuracy of >80% on the test set. As separate classifiers for antrum and corpus were used, we were also able to identify severe inflammation restricted to corpus and severe pangastritis, thereby identifying patients with increased risk for gastric cancer. The results should be interpreted with caution because of the limited number of samples, but they show the great potential of using CNNs for the classification of gastritis. The gastritis subtypes may exhibit a different degree of inflammation and the separation into A, B, and C gastritis does not cover the complete non-neoplastic spectrum of gastric pathologies. Moreover, the classifier may not detect histopathological changes that were not included in the classifier such as, for example, granulomas which may be important for making an appropriate diagnosis. Additionally, neoplastic changes would be missed by our CNN. Furthermore, there may be mixed gastritis types such as a combination of type A and type B gastritis which are a particular challenge for CNN-based classifications. Specifically, cases that were classified by the CNN as type A gastritis with severe inflammation in antrum are a problematic category in a real-life scenario, as they are very likely to represent either type B gastritis with severe atrophy or mixed type A and B gastritis. Using our approach, these cases could be filtered and specifically highlighted, requiring a detailed critical review for final diagnosis. It is important to note that the evaluation of gastric biopsies requires a large body of knowledge and experience in order to detect and correctly interpret the respective histological changes and goes far beyond the categorization into type A, B, or C gastritis. The presence of intestinal metaplasia and atrophy are important independent variables for predicting the risk of gastric cancer and should be mentioned in a histopathological report. Based on the abovementioned statements, it becomes clear that the application of CNN for histopathological classifications must always be conducted under the supervision of a pathologist to avoid misdiagnosis and potentially harmful consequences for patients.

Digital pathology in combination with the application of CNN for the classification of histopathological images has great potential to semi-automate the diagnostic workflow, which is expected to reduce the pathologists’ time per case, especially for high-volume diagnostic tasks such as gastritis diagnostics. This time per case will become a particularly important issue, as there is a deficit forecast for pathologists and an expected increase in the overall case load and quality demand [46,47,48]. Moreover, these digital, algorithm-supported workflows could be beneficial for countries which lack pathological expertise. On the other hand, the use of digital workflows with its needs for additional equipment is not yet adequately reimbursed to the best of our knowledge, and the need for additional expertise in computer technology may also prevent rapid widespread implementation [49]. Moreover, it seems that digital review is equivalent to traditional review of slides, but efficiency might be worse when not used in conjunction with tools that facilitate or automate the review process [50]. In the long term, the ongoing specialization will lead to an unavoidable centralization of histopathological expertise [47]. It is difficult to imagine an alternative non-digital solution to these challenges, and in our opinion, the quality-controlled application of CNNs has great potential to complement the digitalization process.

## 4. Materials and Methods

### 4.1. Patient Cohort and Scanning of Tissue Slides

A cohort of the three most frequent gastritis subtypes, type A (*n* = 49), type B (*n* = 39), and type C-gastritis (*n* = 47) was assembled from the archive of the Institute of Pathology, University Clinic Heidelberg. Diagnoses were made according to the modified Sydney Classification [3]. The study was approved by the local ethics committee (#S-207/2006, and #S315/2020). Hematoxylin- and eosin-stained tissue sections were extracted and scanned at 400× magnification using a slide scanner (Aperio SC2, Leica Biosystems, Wetzlar, Germany).

### 4.2. Region Annotation and Image Patch Extraction

**Figure 6:**
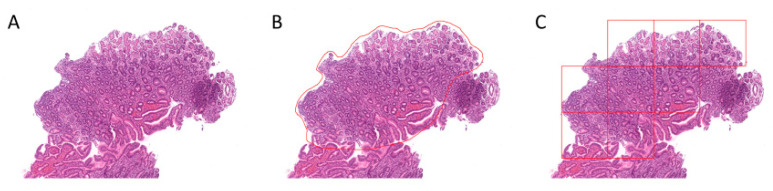
Illustrated process of image patch extraction. A representative example is shown of how image patches were generated within QuPath. The region of interest (**A**) was annotated ((**B**), red outline) and image patches 500 × 500 µm in size were generated ((**C**), red squares).

**Figure 7:**
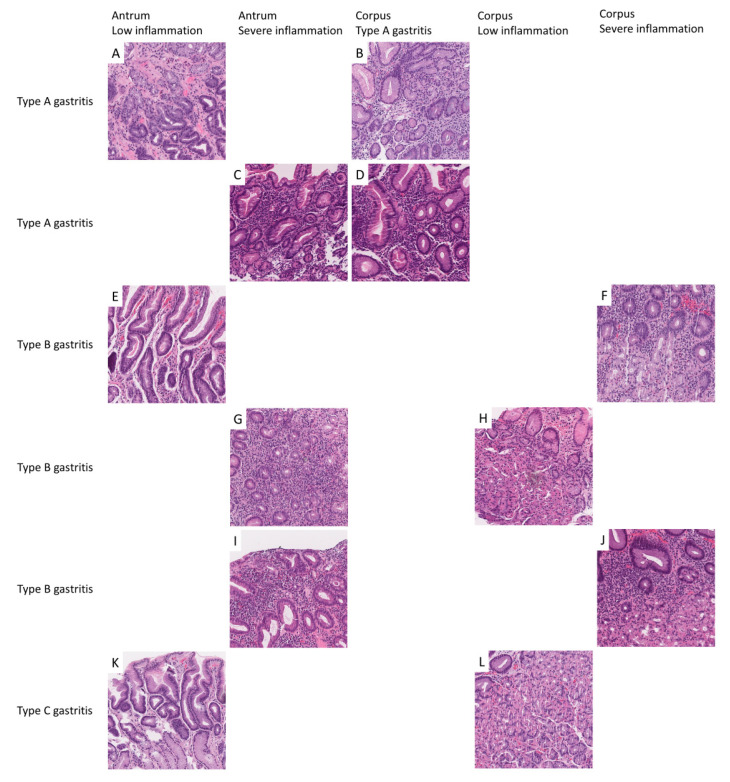
Examples of extracted image patches and the interpretation of the classification results. Representative image patches were extracted from antrum and corpus biopsies from patients with type A (**A**–**D**), B (**E**–**J**), and C (**K**,**L**) gastritis. Image patches exhibit typical histomorphological changes associated with the respective gastritis subtype. Image patches from antrum (**A**,**C**,**E**,**G**,**I**,**K**) were separated into low inflammation (**A**,**E**,**K**) and severe inflammation (**C**,**G**,**I**). Image patches from corpus (**B**,**D**,**F**,**H**,**J**,**L**) were separated into type A gastritis (**B**,**D**), low inflammation (**H**,**J**), and severe inflammation (**F**,**J**).

Scanned slides were imported into QuPath (v.0.2.0-m9); representative areas A, B, and C gastritis were annotated by a pathologist (M.K.); patches 500 × 500 µm (1973 × 1973 px) in size were generated within QuPath; and the respective image patches were exported to the local hard drive for antrum and corpus, respectively [51]. Relatively large patches were chosen to be able to capture representative features of the respective gastritis subtype (Figure 6). Representative image patches from A, B, and C gastritis are displayed in Figure 7.

### 4.3. Nomenclature of Image Patches and Encoding of Diagnosis

Image patches were extracted separately for antrum and corpus. As inflammation was used as a surrogate, type A gastritis can only be diagnosed in corpus, and type C gastritis cannot be diagnosed in corpus, images from patients were allocated within the following categories: LI and SI in antrum and type A gastritis, LI and SI in corpus. LI was defined as none or mild granulocytic or mononuclear infiltrate (according Sydney grade none and mild) and SI was defined as moderate and severe granulocytic or mononuclear infiltrate (according to Sydney grade moderate and severe). If at least a moderate either granulocytic or lymphocytic infiltrate was present, the case was considered SI.

As we were expecting a CNN to perform poorly on images with subtle differences, difficult to separate even for human pathologists, changes that were close to normal were lumped in the LI category and a separate category with normal-appearing antrum and corpus mucosa was not introduced. Moreover, a two-tiered system is more consistent than a three- or four-tiered system. The respective nomenclature is displayed in Table 5 and Figure 7.

### 4.4. Hardware and Software

Our calculations were performed with an AMD Ryzen 7 3700X CPU (Advanced Micro Devices, Santa Clara, CA, USA), 16 GB G.SKILL Ripjaws V RAM (G.SKILL International Enterprise, Taipei, Taiwan), and a GeForce RTX 2070 SUPER (Nvidia Corporation, Santa Clara, CA, USA) graphics card. The following software was used: 64-bit Windows 10 Pro (Microsoft Corporation, Albuquerque, NM, USA). R (v.4.0.1) with RStudio (v.1.3.959, RStudio, Boston, MA, USA) and the R-packages Keras (v.2.3.0.0), generics (v.0.0.2) reticulate (v.1.16-9000), tfruns (v.1.4), magrittr (v.1.5), zeallot (v.0.1.0), R6 (v.2.4.1), tensorflow (v.2.2.0), config (v.0.3), jsonlite (v.1.6.1), processx (v.3.4.2), yaml (v.2.2.1), rstudioapi (v.0.11), caret (v.6.0-86), and e1071 (v.1.7-3). Conda (v.4.8.3) and Python (v.3.6.10) with TensorFlow (v.2.2.0, Google Brain, Mountain View, CA, USA) and numpy (v.1.18.5).

### 4.5. Analytical Subsets

To ensure reliable results, image patches from patients with antrum and corpus were randomly separated into training (60% of patients), validation (20% of patients), and test sets (20% of patients). Image patches from patients with either only antrum or corpus were always assigned to the training set. All image patches from a patient were in one of the sets only. These subsets were not changed during the analyses.

### 4.6. Convolutional Neuronal Networks

For the CNNs, we use the Xception architecture, which uses an extreme version of the inception modules introduced with inception-style models like InceptionV3 [32,52]. Xception models indicate superior performance on classical image classification tasks like ImageNet compared to InceptionV3 models. We did not include a fully connected dense layer before the last logistic layer. We trained all our models for 100 epochs with a batch size of eight. Although our original image size was 500 × 500 µm corresponding to 1972 × 1972 px, we used a fixed image input size of 299 × 299 px in order to be able to fit a sufficient number of images into the GPU RAM. Prior to training, we confirmed manually that the characteristic features could still be identified at a resolution of 299 × 299 px. During training, we varied the learning rate (1 × 10^−4^, 1 × 10^−5^, 1 × 10^−6^, 1 × 10^−7^, 1 × 10^−8^), the dropout rate just before the last logistic layer (0, 0.2, 0.5) and switched between using a regularization of 2 × 10^−5^ or using no regularization.

## 5. Conclusions

In the present study, deep learning was applied to classify gastritis subtypes based on antrum and corpus biopsies. Further studies on larger patient cohorts are necessary to confirm our findings.

## Figures and Tables

**Table 1:** Number of patients and image patches in the training, validation, and test sets.

Set/Region	Antrum Image Patches, *n* (%)	Corpus Image Patches, *n* (%)
Overall (patients *n* = 135, image patches *n* = 1230)	682	548
Training (patients *n* = 62 with both)	(+19 patients with only antrum)	(+11 patients with only corpus)
LI	265 (57)	133 (37)
SI	197 (43)	108 (30)
A gastritis	/	122 (34)
Validation (patients *n* = 21)		
LI	64 (57)	21 (25)
SI	48 (43)	29 (35)
A gastritis	/	34 (41)
Test (patients *n* = 22)		
LI	84 (78)	38 (38)
SI	24 (22)	14 (14)
A gastritis	/	49 (49)

**Table 2:** Antrum and corpus classifier confusion matrix and statistics for the validation image patch set.

	Antrum Classifier		Corpus Classifier		
Confusion Matrix (by Image Patches)	LI Predicted	SI Predicted	LI Predicted	SI Predicted	A Gastritis Predicted
LI true	54	10	27	3	4
SI true	16	32	0	21	0
A gastritis true	/	/	5	16	8
Statistics					
Accuracy (95% CI)	0.77 (0.68–0.84)		0.67 (0.55–0.77)		
Classes	LI vs. SI		LI vs. other	SI vs. other	A gastritis vs. other
Sensitivity	0.77		0.53	0.67	0.84
Specificity	0.76		1.00	0.71	0.87
Positive predictive value	0.84		1.00	0.28	0.79
Negative predictive value	0.67		0.70	0.93	0.90

CI, confidence interval; LI, low inflammation; SI, severe inflammation; vs., versus.

**Table 3:** Antrum and corpus classifier confusion matrix and statistics for the test image patch set.

	Antrum Classifier		Corpus Classifier		
Confusion Matrix (by Image Patches)	LI Predicted	SI Predicted	LI Predicted	SI Predicted	A Gastritis Predicted
LI true	76	8	27	20	2
SI true	8	16	6	26	6
A gastritis true	/	/	7	3	4
Statistics					
Accuracy (95% CI)	0.85 (0.77–0.91)		0.56 (0.46–0.66)		
Classes	LI vs. SI		LI vs. other	SI vs. other	A gastritis vs. other
Sensitivity	0.90		0.53	0.33	0.68
Specificity	0.67		0.77	0.89	0.64
Positive predictive value	0.90		0.68	0.29	0.55
Negative predictive value	0.67		0.63	0.91	0.75

CI, confidence interval; LI, low inflammation; SI, severe inflammation; vs., versus.

**Table 4:** Gastritis classifier confusion matrix and statistics for the test patient set.

	Gastritis Classifier		
Confusion Matrix (by Patient, *n* = 17)	A Gastritis Predicted	B Gastritis Predicted	C Gastritis Predicted
A gastritis true	7	0	1
B gastritis true	1	3	0
C gastritis true	0	0	5
Statistics			
Accuracy (95% CI)	0.84 (0.64–0.96)		
Classes	A vs. other	B vs. other	C vs. other
Sensitivity	0.88	1.00	0.83
Specificity	0.89	0.93	1.00
Positive predictive value	0.88	0.75	1.00
Negative predictive value	0.89	1.00	0.92

Five patients from the test set with ties not included. CI, confidence interval; vs., versus.

**Table 5:** Nomenclature of the extracted image patches and encoding of gastritis diagnosis.

Overall Gastritis Diagnosis	Antrum Finding/Classifier Result	Corpus Finding/Classifier Result
A	SI	A gastritis
B	SI	SI
B	SI	LI
A	LI	A gastritis
B	LI	SI
C	LI	LI

LI, inflammation; SI, severe inflammation.

## Supplementary Materials

The following are available online at https://www.mdpi.com/1422-0067/21/18/6652/s1.
