# Supplementary material for: Identification of Gastritis Subtypes by Convolutional Neuronal Networks on Histological Images of Antrum and Corpus Biopsies
Source: Int J Mol Sci. 2020 Sep 11;21(18):6652. doi: 10.3390/ijms21186652 (PMC7555568; doi:10.3390/ijms21186652)
Supplement: Supplementary file 1 [file ijms-21-06652-s001.pdf]

Supplementary Materials

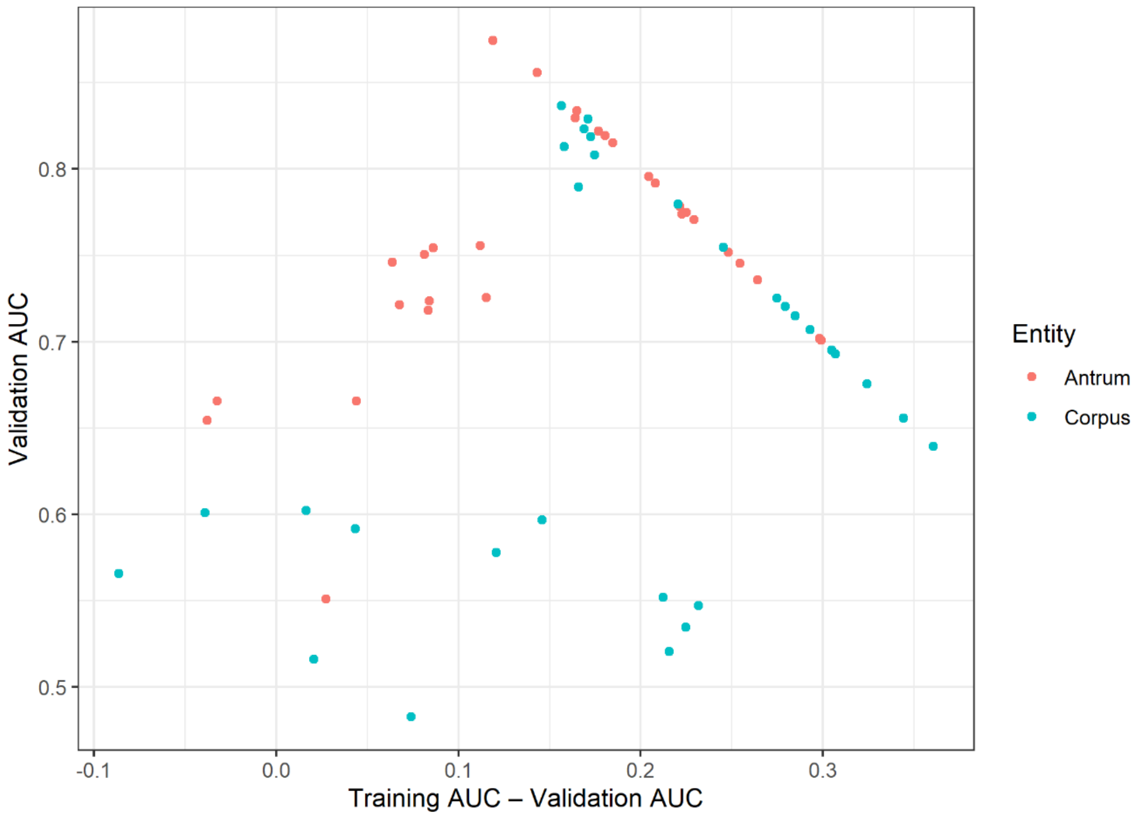

Supplementary Figure 1: Validation AUC and extent of overfitting.

For each trained model on antrum and corpus image patches the validation AUC and the extent of overfitting (training AUC minus validation AUC) is shown. Displaying overfitting, particularly in the good models with high validation AUC, a certain degree of overfitting is observed – indicating that the trained models do not generalize fully in the validation set.

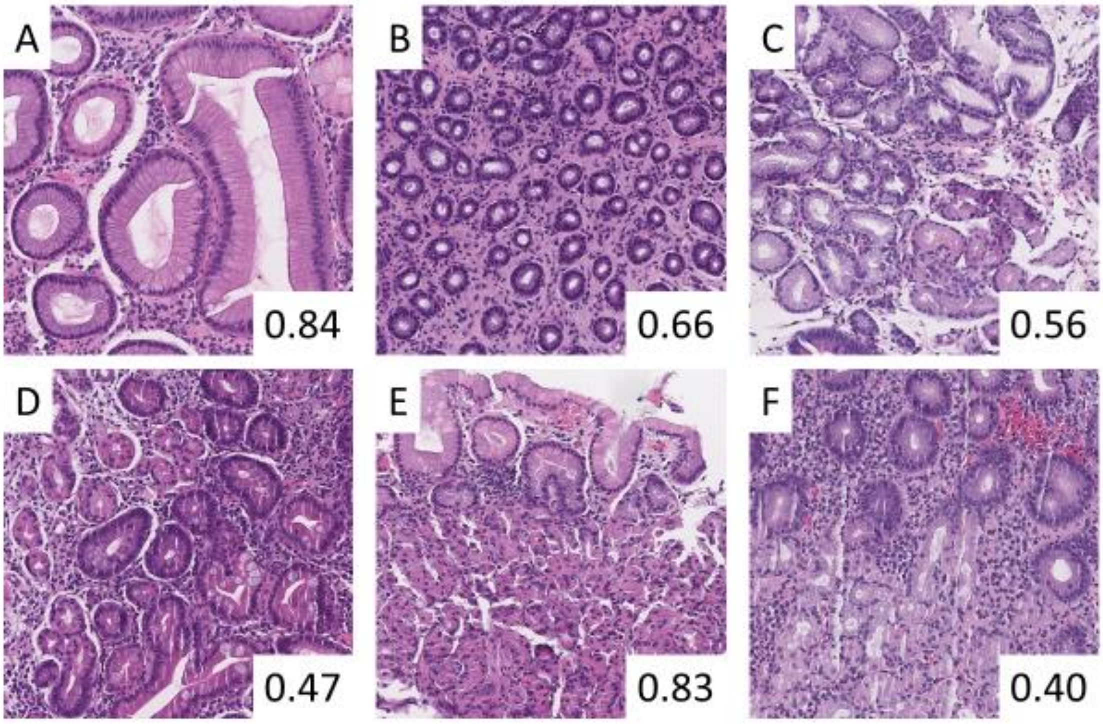

Supplementary Figure 2: Examples of misclassified image patches.

The figure shows examples of misclassified images patches. Classification probability for the respective misclassified class is displayed in the right corner. Although not completely justified, as it is not known, how the model comes to a classification result, the selected misclassifications seem explainable when reevaluated by a pathologist. In this regard, images judged as having a rather high content of inflammatory cells (SI) may be classified as having a low inflammation (LI) because other structures such as a large foveolar gland make up a large portion of the image (A). As all image from one patient were considered for the judgement of LI or SI it may also happen that single cells have a low to moderate content of inflammatory cells but are classified as SI (B). With regard to type A gastritis (C, D) it seems plausible that relatively small features such as parietal cells or intestinal type epithelium which account for <5% of the image, may be missed by the classifier, especially when the algorithm was trained on a small dataset. Also, if parietal cells appear to be homogeneous or sparse, images that would truly be LI (E) or SI (F) may also be misclassified as type A gastritis.
